# Prospects and recommendations for risk mapping to improve strategies for effective malaria vector control interventions in Latin America

**DOI:** 10.1186/s12936-015-1052-1

**Published:** 2015-12-23

**Authors:** Temitope O. Alimi, Douglas O. Fuller, Martha L. Quinones, Rui-De Xue, Socrates V. Herrera, Myriam Arevalo-Herrera, Jill N. Ulrich, Whitney A. Qualls, John C. Beier

**Affiliations:** Abess Center for Ecosystem Science and Policy, University of Miami, Coral Gables, FL USA; Department of Geography and Regional Studies, University of Miami, Coral Gables, FL USA; Department of Public Health, Universidad Nacional de Colombia, Bogota, Colombia; Anastasia Mosquito Control District, 500 Old Beach Road, St. Augustine, FL USA; Centro de Investigacion Cientifica Caucaseco, Universidad del Valle, Cali, Colombia; School of Health, Valle State University, Cali, Colombia; Department of Public Health Sciences, Miller School of Medicine, University of Miami, Miami, FL USA

**Keywords:** *Anopheles*, Vector control, Mosquito ecology, Malaria elimination, Environmental changes, Risk mapping, Latin America

## Abstract

With malaria control in Latin America firmly established in most countries and a growing number of these countries in the pre-elimination phase, malaria elimination appears feasible. A review of the literature indicates that malaria elimination in this region will be difficult without locally tailored strategies for vector control, which depend on more research on vector ecology, genetics and behavioural responses to environmental changes, such as those caused by land cover alterations, and human population movements. An essential way to bridge the knowledge gap and improve vector control is through risk mapping. Malaria risk maps based on statistical and knowledge-based modelling can elucidate the links between environmental factors and malaria vectors, explain interactions between environmental changes and vector dynamics, and provide a heuristic to demonstrate how the environment shapes malaria transmission. To increase the utility of risk mapping in guiding vector control activities, definitions of malaria risk for mapping purposes must be standardized. The maps must also possess appropriate scale and resolution in order to become essential tools in integrated vector management (IVM), so that planners can target areas in greatest need of control measures. Fully integrating risk mapping into vector control programmes will make interventions more evidence-based, making malaria elimination more attainable.

## Progress of malaria control in Latin America

Malaria transmission in Latin America, including Central America, the Caribbean, and South America is a persistent problem. With highly focal malaria, about 120 million people in Latin America are at risk, out of which an estimated 25 million people are at high risk of malaria transmission [1, 2]. Malaria risk has no standard definition, with risk being defined according to the subject of interest. However, generally in public health, risk is defined as ‘the probability of disease developing in an individual in a specified time interval’ [3]. Malaria risk is estimated by the World Health Organization (WHO) based on annual parasite index (API), the number of positive parasite slides per thousand population. On this basis, most of the at-risk population in Latin America live in low transmission settings where cases are ≤1 per 1000 (see Table 1), the rest live in high transmission areas with >1 case per 1000 [4]. The spatial distribution of infections is heterogeneous with the majority caused by infections by *Plasmodium vivax*, which accounts for about three-quarters of all cases in the region; whereas, *Plasmodium falciparum* is exclusively responsible for the infections in parts of the Caribbean (Haiti and Dominican Republic), most of the infections in the Guyana Shield (French Guiana, Guyana and Suriname) and along the Pacific coast of Colombia [1, 2, 4]. The burden of malaria is also widely disparate. Approximately 90 % of the malaria burden of the region is borne by countries in the Amazon Rainforest [5]; three countries accounted for 72 % of cases in 2013: Brazil (42 %), Colombia (12 %), and Venezuela (18 %) [2].

**Table 1:** Malaria burden in the Americas in 2013

Sub-region	Country	Level of transmission (percentage of population)			Disease burden (percentage of cases)	
		Malaria free	Low (≤1 per 1000)	High (>1 case per 1000)	*P. vivax*	*P. falciparum*
Central America	Belize	30	70	0	100	0
	Costa Rica	62	37	1	66	4
	El Salvador	30	70	0	97	3
	Guatemala	0	14	86	98	2
	Honduras	27	59	14	84	16
	Mexico	97	3	0	99	1
	Nicaragua	50	49	1	82	18
	Panama	24	71	4	100	0
Caribbean	Haiti	0	47	53	0	100
	Dominican Republic	14	81	4	0	100
South America	Bolivia	65	30	5	90	10
	Brazil	80	18	2	83	17
	Colombia	78	8	15	72	28
	Ecuador	40	59	1	75	25
	French Guiana	0	14	86	68	32
	Guyana	7	58	35	38	62
	Paraguay				38	
	Peru	84	12	5	84	16
	Suriname	84	0	16	55	45
	Venezuela	81	16	3	70	30

Source: World Malaria Report 2013 [1] and 2014 [2]

Nevertheless, malaria control has vastly improved in the past decade. Confirmed malaria cases fell from 1.2 million to 427,000 cases from 2000 to 2013, while deaths from malaria declined from 390 to 82 deaths [2]. Although Guyana and Venezuela recorded increased incidence in cases in 2012 [1, 2], two countries in South America (Chile and Uruguay), and all Caribbean countries except Haiti and Dominican Republic, are malaria free. Thirteen countries recorded 75 % or greater decline in malaria incidence between 2000 and 2013 [2]. Progress toward elimination is also ongoing in many of the low-transmission settings in the region. Seven countries (Argentina, Belize, Costa Rica, Ecuador, El Salvador, Mexico, and Paraguay) are currently in the pre-elimination phase [2], a stage in which malaria control is firmly established and access to preventive measures, diagnostic testing and treatment is available to the population at risk [6].

The observed progress is facilitated by improvements in malaria surveillance and monitoring, more efficient use of control measures, prompt and efficient malaria treatment/drugs, and better integrated vector management (IVM) implementation [7]. IVM is a rational decision-making approach for optimizing the use of resources for vector control by involving the adaptation of strategies and interventions based on local vector ecology and epidemiology [8]. Confirmation of malaria cases is expedited through routine malaria surveillance, and countries such as Mexico, Ecuador, Costa Rica and Paraguay implemented intense case surveillance [9, 10]. The range of diagnostic testing and reporting also expanded, a development that may account for the increased reported malaria cases in Venezuela and Haiti, rather than an actual increase in malaria incidence [2, 9]. Long-lasting insecticidal nets (LLINs), indoor residual spraying (IRS) or both are now applied for at-risk populations in all countries with ongoing malaria transmission [2]. Six countries (Bolivia, Mexico, Guatemala, Nicaragua, Ecuador and Costa Rica) have more than 50 % of populations at high risk covered with LLINs and IRS [1, 2, 9]. Antimalarial drugs are also sufficiently available for all patients who seek treatment in public health centres [2]. These marked improvements in malaria control have been made possible mainly by international (e.g. Global Fund, President’s Malaria Initiative and World Bank) and domestic funding, which increased from US $153 million in 2005 to US $214 million in 2011, but dropped to US $140 million in 2013 [2]. Regional collaborations (e.g. Malaria Control Programme in Andean-country Border Regions, the Amazon Malaria Initiative and the Amazon Network for the Surveillance of Antimalarial Drug Resistance) have also been instrumental in developing drug efficacy protocols and monitoring drug resistance [7, 9]. These achievements indicate that malaria elimination in Latin America is feasible if current efforts are strengthened and new interventions are developed and implemented.

The strategies needed to achieve malaria elimination are multi-pronged and require different approaches. There is a need to explore how using novel methods incorporated in risk mapping/modelling, can enhance the progress already accomplished. Risk mapping is a methodology that provides spatial detail on the expected distribution of vectors or risk of exposure to malaria and help identify underlying factors, which contribute to risk and burden [11]. Strengthening current vector control and developing new tools cannot be over-emphasized in the drive towards elimination. Spatially accurate, high-resolution risk maps that delimit areas of likely human-vector contact can guide IVM implementation and thus should be considered a priority. This review focuses on how current vector control strategies in Latin America can be improved using novel methods in risk mapping to enhance elimination programmes.

## Constraints of malaria vector control in Latin America

Despite appreciable progress, the final steps toward malaria elimination in the region are challenging, yet surmountable. Differences in epidemiology coupled with the geography of each country determine the kind of malaria intervention required in a particular region and its efficacy [1, 2]. The unique Latin American landscape for the most part favourably predisposes the region to malaria elimination compared with other regions where malaria is endemic e.g. Africa and South-east Asia, if efforts are effectively tailored. For instance, the altitudinal gradients of Latin America are greater than in Africa or Southeast Asia, with the Cordillera that runs from Mexico to Chile serving as a major barrier to transmission and vector dispersal. Location of settlements in Latin America is also determined largely by access through river networks [12], as opposed to that in Africa, where spread and movement of people and parasites is more porous because much of the transportation is land-based [13]. Moreover, vast areas of savanna and semi-arid lands are interconnected in Africa [14] whereas in Latin America, because of the highly focal nature of the disease, geographic isolation of the Amazon and other low-lying zones, the areas where population are at risk are much more easily delineated. These geographical advantages can be manipulated through risk mapping to broaden knowledge of malaria epidemiology and mosquito ecology in Latin America and thus help to strengthen malaria control. However, despite this advantage, other factors discussed in the next section limit malaria elimination in the region.

## Limited entomological capacity

Despite the improved entomological capacity evident in Latin America, there still remains a shortage of skilled entomologists and entomological infrastructure [15, 16]. Well-trained entomologists and fully equipped and functioning insectaries and entomological laboratories are necessary for targeted vector control and vector surveillance, yet the capacity to fully execute these are limited [15]. As a result, there are few transmission studies and investigations of outdoor-resting and early evening biting (behavioural traits of Latin American vectors that could confound current vector control gains) [17–20]. National needs assessments of current capacity are needed and such survey data can also be mapped to indicate where more investment in training and laboratory infrastructure is needed.

Risk mapping methodologies can to a large extent address the limitations in current entomological capacity for malaria control in Latin America. The methodologies provide insights into pathogen transmission dynamics, including knowledge about the transmission and endemicity of parasites. For instance, Patil et al. [21] used Bayesian geo-statistics (BG) to produce candidate maps of *P. falciparum* endemicity in Africa. Bayesian geo-statistics accounts for the spatial variability inherent in a dataset by finding the unknown true map from a large sample of maps that reflect the dataset [21]. The approach was similarly used by Gething et al. [22, 23] to map global *P. falciparum* and *P. vivax* endemicity respectively. Using georeferenced parasite rates and incidence data, a continuous surface showing transmission intensity for *P. falciparum* was created [22]. For *P. vivax* mapping, georeferenced age standardized *P. vivax* parasite rates were incorporated with climatic factors (temperature and aridity) and medical intelligence data to produce maps of *P. vivax* endemicity [23]. The method is particularly useful in estimating risk in areas with limited data and has the added advantage of accounting for uncertainties in the results. It is however noteworthy that maps generally report single estimates for each location without conveying the variability inherent, even when data are evenly distributed, e.g. in un-sampled locations [21], an important limitation, which may preclude the widespread use of risk modelling.

While exploring risk of malaria transmission based on outdoor and early- biting mosquitoes is newly developing, risk-modelling methodologies can provide necessary tools for mapping locations, distribution and effects on transmission of exophilic and exophagic mosquitoes and direct vector control efforts geared towards them. For example, using an individual-based simulation model, Griffin et al. [24] showed that very high coverage of current vector control interventions (>90 %) or development of new control measures are necessary to reduce *P. falciparum* transmission in high transmission areas of Africa where outdoor biting, *An. arabiensis* prevail, but similar studies utilizing risk modelling have yet to be conducted in Latin America. This is probably due to the limited knowledge about outdoor vectors species in the region, limited coverage and knowledge of the effectiveness of current vector control tools, as well as limitations in human resources.

## Increasing insecticide resistance

Growing insecticide resistance among malaria vectors in Latin America has raised concern, yet data on insecticide resistance are sparse [25, 26] and their mapping limited. Resistance to dichlorodiphenyltrichloroethane (DDT), pyrethroids and organophosphates (OP) has been observed in parts of the region. For instance, in Colombia, resistance to DDT occurs in *Anopheles darlingi* around Quibdo and the Atrato River [27, 28] and resistance to pyrethroids exists for both *An. darlingi* and *An. albimanus* in Chocó [29]. Mild cross-resistance to DDT and pyrethroids, and high resistance to pyrethroids and OP occurs in populations of *An. nuneztovari* in parts of Colombia [30]. In Peru, *An. albimanus* is resistant to pyrethroids [31] while relatively lower resistance to OP and pyrethroids and high resistance to DDT occur in the same species in southern Mexico [32]. Laboratory colonies of *An. albimanus* from Guatemala show resistance to DDT and pyrethroids, whereas field species from El Salvador and Belize were susceptible to these two insecticides [33]. Although resistance of mosquitoes to insecticides may be more widespread in this region than has been reported, the limited data available provide opportunities for mapping insecticide resistance and risk associated with it, so efforts to tackle the problem can be better targeted.

Risk mapping and modelling technologies have capabilities for describing the distribution of insecticide resistance in mosquitoes. Coleman et al. [34] used a malaria information system equipped with geographic information system (GIS) capability to map locations of insecticide resistance across Africa. Geo-referenced resistance information was obtained from published reports to show a spatial distribution of resistance across the African continent. Additionally, Insecticide Resistance (IR) Mapper, an online mapping tool, has also been developed to examine spatio-temporal trends in *Anopheles* resistance using geo-referenced data [35]. Incorporating insecticide resistance and susceptibility data from various countries, the developers conclude that the IR mapper would aid visualization and direct vector control through insecticide applications. However, such mapping technologies are data-driven, depending on accurately geo-referenced insecticide resistance information, which may be limited in developing countries. Thus, although the facilities to map and display mosquito resistance to insecticides are now available, more studies documenting insecticide resistance, including accurate locational information in different parts of Latin America are required. It is particularly necessary to have a routine system of insecticide resistance surveillance in Colombia, Honduras, Nicaragua and Brazil, where mass distribution of LLINs is currently promoted and implemented in high-risk areas [1, 2].

## Inconsistent policy implementation and monitoring of programme efficacy

Vector control in Latin America is increasingly implemented based on principles and policies of IVM, but operations are yet to be consistently employed throughout the region. Being evidence-based, the IVM approach advocates that vector control interventions be introduced and implemented based on prior information [8]. However, this does not always happen in practice. In an assessment of malaria control strategies conducted in Ecuador, Peru, Colombia, Bolivia and Guyana, Flores et al. [16] observed that information on research, extent and quality of IRS application was incomplete. Likewise LLINs were administered without prior studies to determine target populations, as well as understand vector behaviour or their response to insecticides in all the countries except Bolivia and Colombia [16, 36].

Whereas coverage and delivery of vector control tools to populations has greatly improved in the past decade, strict compliance with technical guidelines on the tools are still lacking. For instance, Flores et al. [16] found that WHO guidelines for IRS applications were not strictly followed. Moreover, target populations in remote areas live in houses that do not conform to technical criteria for IRS spraying. Although LLINs were delivered, delivery was sometimes diverted to areas not targeted for bed nets [16]. Coverage of entire target populations also fell below the 80 % coverage criteria for LLINs [16]. Furthermore, other vector control measures such as environmental management and mosquito proofing of houses are not as widely used especially in remote areas. Evaluation of application strategies is also sporadic [37, 38]. For example, assessment of the efficacy of interventions, timeliness and frequency of applications for IRS or insecticide resistance are limited [14, 39].

Parts of the policy implementation issues arising from lack of information can be improved through risk modelling. Risk maps can provide prior knowledge on target (at-risk) populations and their stratifications [40–42], information that can help identify specific locations for prioritization of malaria interventions. For instance, Tatem et al. [40] used spatial models to improve estimates of children under 5 at highest risk of *P. falciparum* transmission in Tanzania. Noor et al. [42] also mapped population distribution, stratifying by age, in order to estimate malaria risk and quantify coverage of interventions. This is analogous to the application of remote sensing (RS) in precision agriculture to help farmers better target where pesticides, herbicides, fertilizers, water, etc. are needed within their fields [43, 44]. This has allowed farmers save millions of dollars by targeting only those areas that need inputs the most (i.e., pesticides, etc.) rather than broadcasting chemicals indiscriminately [43, 44]. Maps containing information on housing location and type [45, 46] can also provide knowledge which together with information on target population and vector distribution can guide targeted LLIN and IRS applications [40–42]. In some villages in northern Sri Lanka, Van Der Hoek et al. [47] found proximity of housing to vector breeding sites and poor housing construction major risk factors for malaria in the area. Information on vector behaviour and distribution [17, 47, 48] and about insecticide resistance [35] is also enhanced through risk mapping. Such information gives an indication of where vectors can be found and targeted. For example, a number of studies such as Sinka et al. [17] and Fuller et al. [48] used species distribution model (SDMs) to map the distribution of dominant anophelines in the Americas and current *An. albimanus* distribution in Meso- America respectively. In the latter, the mosquito data was extrapolated to a future period, thus reducing the impact of sampling bias on the data, an implication which may have more of an impact on policy.

Risk mapping methodologies also have capabilities to generate information on monitoring malaria interventions e.g. the effect of continued dissemination of LLINs, IRS, mass drug administration and future vaccine on malaria transmission [24] or keeping track of vector control coverage e.g. IRS application [49]. Malaria surveillance, especially in areas with limited resources, is also enhanced through risk mapping. Combining satellite imagery, mobile phone call records and surveillance data in Namibia, Tatem et al. [50] showed that the maps produced could help track and contain malaria, by limiting exported cases and directing efforts in areas with imported cases. Routine mapping of malaria incidence or prevalence [51, 52] and targeting hotspots of transmission [53–55] are also strengthened through risk mapping. Bousema et al. [54] elucidated on the spatial patterns of malaria transmission in northeastern Tanzania, identifying hotspots of transmission through clusters of higher malaria incidence created using geo-referenced malaria incidence and mosquito sampling data. De Castro et al. [56] also used spatial modelling to identify clusters of malaria and patterns of transmission in one of the colonization areas in Brazil, highlighting their utility in targeted malaria control.

To monitor progress of malaria control and actualize malaria elimination, risk mapping efforts in Latin America and elsewhere need to focus more on the geo-referencing of implementation of specific intervention strategies as a way to better understand why transmission persists in some places and not in others. This process of ‘efficacy mapping’ involves the mapping of control efforts (either through investments of resources, training, or implementation e.g., distribution of LLINs) relative to outcomes, which are regularly monitored and mappable e.g. incidence through time. This kind of mapping has the potential to greatly enhance elimination efforts in Latin America and elsewhere. However, the difficulties associated with the implementation and monitoring of policies in the region are not limited to information deficits, but also related to human capacity and infrastructural deficiencies, corruption, as well as political will, all factors that risk mapping may not be readily able to address. Furthermore, if efficacy remains low in the face of sustained investment in control measures, it may signal lack of institutional capacity or will to achieve elimination.

## Varying definitions of risk and measurement methods

Risk assessment is an important component of public health, which provides information that may aid decision-making either for the public or public health agencies [57]. Yet, there is currently no standard definition of risk, rather it is described based on the subject of interest. Risk is defined broadly by the Society for Risk Analysis as ‘the potential for realization of unwanted, adverse consequences to human life, health, property or the environment’ [58]. In public health, risk refers to ‘the probability of disease developing in an individual in a specified time interval’ [3]. Definition of risk becomes increasingly varied for vector-borne diseases, such as malaria, because of the complexity of the disease [59] and is, therefore associated with variables related to both the disease and its vectors (See Table 2). Thus in the context of mapping, ‘malaria risk’ may be considered an array of factors that relate not only to the presence and density of vectors and parasites, but also to the level of investment and implementation of different malaria control measures, which vary greatly in space and time. Maps based on repeatable, reliable measurements (e.g., those based on remote sensing) provide a basis for visualizing changing risk landscapes that are inherent in many parts of Latin America. Malaria risk could also be considered in terms of trends over time, which could be estimated from time series data and displayed in map form as a multi-year trend. The time series data, which are available for many countries in Latin America may be disaggregated to the municipal level, and could represent an innovation in malaria risk mapping.

**Table 2:** Some definitions of malaria risk and types of risk mapped

Reference	Definition of risk	Study area	Type of risk mapped
Chaparro et al. [52]	Current malaria incidence and prevalence		
Noor et al. [139]	Probability of plasmodium presence		
Zeilhofer et al. [60]	Habitat suitability		
Fuller et al. [61]	Vector exposure		
Sinka et al. [17], Fuller et al. [48]	Vector presence		
Catillo-Salgado [129]	Intensity of transmission		
Foley et al. [130], Rubio-Palis et al. [131], Manguin et al. [134]		Neotropics	Vector distribution and density
Loaiza et al. [132]		Panama	
Osborn et al. [133], Berti et al. [137]		Venezuela	
Roberts et al. [107], Rejmankova et al. [67]		Belize	
Savage et al. [135], Rodriguez et al. [136]		Mexico	
Mekuria et al. [138]		Dominican Republic	
de Castro et al. [56, 118]		Brazil	
Gething et al. [22, 23], Hay et al. [47]		Global (including the Americas)	Parasite rates and prevalence

The measurement of risk is also as widely varied as its definition. Risk may be estimated using various modelling methods (biological or statistical), explanatory variables (depending on the etiology of the disease and how well this is established) or mapped at different scales and resolutions [59]. Biological models use variables that represent important biological pathways of the infection in modelling risk e.g. including temperature in malaria modelling [21], or hypnozoites in modelling *P. vivax* [62]. Statistical models on the other hand seek statistical associations between the variable of interest (e.g. malaria cases) and its predictors based on the epidemiology of the disease [17, 61]. The scale e.g. continental, regional, national or local, and the spatial resolution i.e. the size of the smallest possible feature that can be seen on an image, at which malaria risk is represented are also important considerations. Available maps of malaria risk in Latin America such as is produced by the Pan American Health Organization (PAHO) are highly generalized, and aggregated at scales which do not allow for meaningful application [63]. They are also of low resolution and delineated according to political boundaries [63]. The variable definitions, conceptualizations, and measurements of risk limit the application of risk maps because there is no risk-mapping standard for malaria.

Considering that they provide consistent measurements of environmental factors associated with vector dynamics, remote sensing provides a viable means of estimating risk and how risk factors change through time. RS technologies are used for malaria vector mapping and malaria case mapping. Vector mapping involves estimation of malaria risk using mosquito location data [17, 61] while risk is estimated in malaria case mapping using actual malaria incidence or prevalence data [52]. Risk is assessed in both cases by combining those data with environmental and socio-economic factors, which favour mosquitoes and malaria [61, 64, 65]. However, the choice of approach is dependent on the availability of geo-referenced data, which is still restricted to small areas or to aggregated state or county level data in many countries of Latin America [61].

Risk modelling tools available in GIS and RS are efficient for the mapping and analysis of disease distribution and variation, and of environmental elements that may predict or explain these variations [66]. With RS, environmental information, such as vegetation density, location of water bodies and water quality [67], presence of submerged and emergent vegetation in wetlands (aquatic macrophytes, AM) [68, 69], presence and density of settlements [67], including impervious surface area and bare soils [70], which can be correlated with risk of vector-borne diseases are extracted from images. These are potentially important risk factors in different context that sum in different ways to create composite risk or overall risk for any given location, which is represented by a pixel on an image. Such images are captured on earth features and climatic factors, through instruments placed on satellites [71]. These instruments record the interaction of earth surface features with radiant energy in different wavelength bands.

**Fig. 1:**
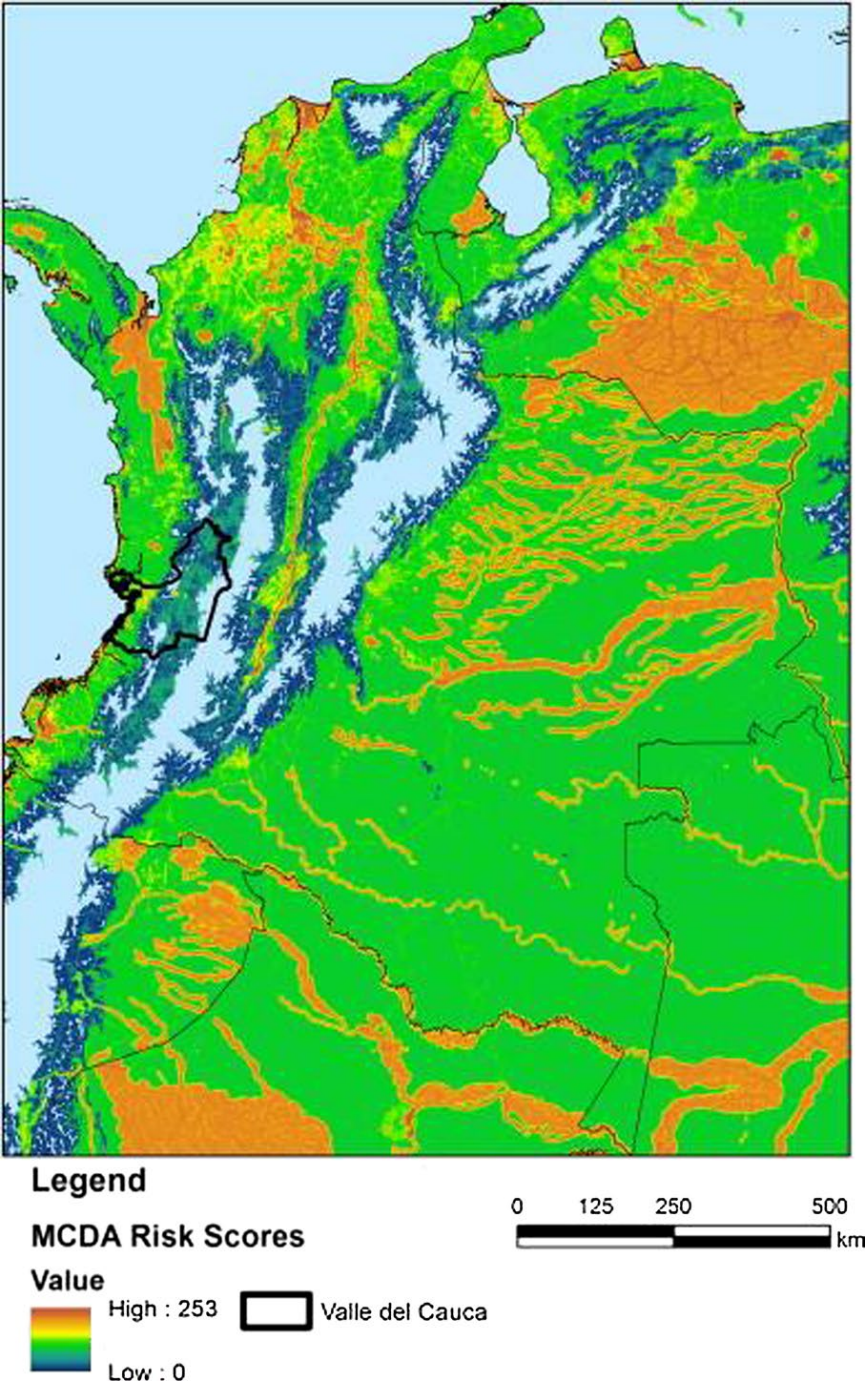
Map of relative risk of exposure to malaria vectors derived from multi-criteria decision analysis (MCDA) guided by expert opinion (EO) in Colombia, parts of Ecuador, Venezuela, Peru and Brazil [61]. Areas in *red* denote high relative risk, areas in *green*, moderate risk, and the areas in *blue* low relative risk of malaria vector exposure

The mapping capabilities provided by high to medium resolution satellite imagery enable improved targeting of areas and populations at risk, so that risk may be reduced [72]; abilities which aid efficient direction of control efforts in both endemic and epidemic situations [73]. The technologies have proven useful in mapping malaria risk in different parts of the world e.g. mapping global *P. falciparum* [22] and *P. vivax* [23] endemicity, mapping dominant *Anopheles* vectors globally [47], in the Americas [17], or in specific countries e.g. Belize [67–69] (See Table 2). The issues of scale and resolution are also effectively handled through risk mapping, as high resolution remotely sensed data are increasingly made publicly available. This has led to the generation of high quality and very fine resolution risk maps, which provide more spatial detail that can aid targeted vector control [61]. By combining knowledge of interactions between vectors, environmental factors and malaria epidemiology, maps of malaria risk may also be generated even if empirical data on malaria distribution are not abundant [61] (See Fig. 1). However, the high cost of fine-resolution RS images, inadequate training in GIS and RS methodologies of health department staff, especially in developing countries, as well as limited understanding of the applications by decision-makers [74] limit their widespread use in many Latin American countries.

## Need to sustain investments and bolster political will to achieve malaria elimination

The political climate influences vector control continuity and progress. Domestic government spending on malaria control in many Latin American countries increased from approximately US $130 million in 2005 to US $160 million in 2011, contributing to the gains earlier mentioned [2]. Yet, unsustained political will and determination of some governments (at least at the local level) and the constantly changing power (decision-makers and power grids) sometimes slow down vector control. Government bureaucracies, which are frequently guided by donor priorities and policies, may be responsible for delays in programme implementation; while, local corruption may also lead to uneven application of malaria control measures [75]. In many countries, ministries of health (MoH) and malaria control programmes are frequently reorganized and their activities decentralized during health sector reforms [76, 77] e.g. the reorganization of the project for control of malaria in the Amazon basin during the Brazilian health sector reform [78, 79].

Brazil has however demonstrated that government commitment to elimination is feasible. In 1993, the country changed the malaria control strategy to focus efforts on high-risk municipalities through early case detection and management [78–81] and more selective use of IRS and environmental management [79], thereby focusing more on individuals than the environment [82]. The MoH increased the number of health posts able to carry out diagnosis and treatment of malaria so that by 2009, there were approximately 3500 diagnostic laboratories, about 50,000 malaria control agents and 2.8 × 10^6^ blood examinations were conducted [83]. Surveillance, monitoring and evaluation activities were also strengthened through the management of Sistema de Informação de Vigilância Epidemiológica (SIVEP-Malaria), the malaria information system for the nation [80]. The result of the concerted efforts was a sustained decrease in malaria cases, disease severity and number of municipalities at risk of malaria in the Amazon [80]. Although Brazil is ahead of its Amazonian neighbours, their systematic approach could/should be used as a model for countries, such as Bolivia, Guyana, Suriname, Venezuela, where malaria control is still in its early stages.

Financial investments in malaria control have started to decline. In 2013, domestic funding decreased to about US $110 million [2]. If this reduction continues, it would become increasingly difficult to maintain the gains in malaria control already achieved. To avert such a situation, investments at the national and local levels must be sustained and governments encouraged in the pursuit of malaria elimination in their territories. Expenditures on malaria control at the subnational level need to be mapped so that political factors can be considered more explicitly and outcomes (e.g., reduced incidence through time) can be matched to investment. To facilitate this, the programme on eliminating malaria in Mesoamerica and Hispanola by 2020 (Eliminación de Malaria en Mesoamerica y la Isla Española (EMMIE)) was initiated by the Global Fund for Aids, Tuberculosis and Malaria (GFATM) and supported by participating countries [84]. The initiative was launched to encourage transition from malaria control to elimination, foster collective action among the countries and bolster sub-regional and political commitment towards elimination [84].

Delivery efficiency [85] for vector control programmes can be enhanced through risk mapping techniques. Mapping transportation routes and human population movements [50], or geographical distribution of interventions delivered through community health workers [85] can help improve distribution channels and give an indication of how effective interventions are. Going forward, by investing in research, which advances the use of risk mapping methodologies in their countries, national and regional governments will produce more value for every dollar spent. This is because risk mapping will guide targeted vector control [40–42], and invariably lead to more efficient use of resources for malaria control. Moreover, mapping things such as investment in control measures per capita, distribution of LLINs per capita, density of health clinics per municipality, degree of spatial isolation, etc. (information which are readily available through national census data and can be realized quickly) that require mapping but have not yet been made spatially explicit can accelerate drive to achieve malaria elimination by highlighting areas where risk remains persistent through time in the face of sustained investment in control measures.

## Gaps in the understanding of vector ecology

The high incidence of *P. vivax* infections, and the different species of vectors and their behaviours make malaria transmission in Latin America unique. Experience based on past control efforts shows that interventions cannot be applied universally regardless of the local environment. There are currently major gaps in understanding distribution, ecology, behaviour, and vector competence of the primary vectors of malaria in Latin America, namely, *An. darlingi*, *An. nuneztovari*, *An. pseudopunctipennis*, *An. albimanus*, and *Anopheles aquasalis* [7, 37, 86]. These gaps in knowledge limit the ability of health authorities to apply adequate vector control measures [7]. With low rates of transmission, government commitment, and fewer residents at risk, relative to other malaria regions, Latin America would appear to be the most feasible location for malaria elimination [37, 87]. However, final steps toward elimination require decreasing the number of infective bites per person to less than one per year [88]. Unfortunately, current vector control strategies in Latin America do not cover the full range of environmental conditions where mosquito exposure occurs, and the existence of even a small percentage of mosquitoes that rest and bite outdoors may prevent the transition from pre-elimination to elimination [89]. This, coupled with the lack of entomological expertise and laboratories equipped with trained personnel to identify vectors and parasites, remains an impediment to elimination.

Indoor residual spraying and LLINs are currently the principal vector control tools in Latin America. IRS use in Latin America began with the introduction of DDT for malaria control in Venezuela [90]. Earlier studies conducted by Gabaldon [90, 91] reported the successful elimination of malaria in most parts of Venezuela using IRS, especially in areas where *An. darlingi* and *An. albimanus* were the main vectors. However, the feeding and resting preferences of most vectors make them poor candidates for control using these tools. IRS targets endophilic mosquitoes while LLINs target anthropophilic, night-biting mosquitoes; characteristics not commonly exhibited by Latin American anophelines. The vectors in Latin America are primarily exophilic, although the degree of exophily varies by region [17, 18]. *An. darlingi* is the main vector, feeding during sleeping hours [19, 20]. *An. albimanus* also exhibits late-night biting and indoor feeding preference [20]. Exophily is however not uncommon with both species as early evening and outdoor biting has been observed [25, 26, 86], as is the case with other species, such as *An. nuneztovari* and *An. pseudopunctipennis* [90]. Despite evidence that these behaviours allow Latin American vectors to evade insecticide exposure with IRS and LLINs, both measures are still the main tools for malaria vector control [20, 92]. It is unclear whether these measures may promote behavioural changes of the vectors (irritability, exiting, or feeding inhibition) and thus contributes to more sporadic malaria transmission in Latin America [2].

Knowledge about the range and distribution of mosquito vectors that transmit malaria is important to guide vector control strategies and provide information that may help prevent future malaria outbreaks. This information has progressively become available through SDMs, which are important tools in the risk-mapping arsenal. SDMs integrated with GIS mapping techniques has seen wider applications in vector mapping in recent years [60, 93, 94] using different modelling applications e.g. boosted regression trees (BRT) [17, 95], and MaxEnt [70, 96]. Extensive use of these tools in Latin America is essential to fill the gaps in knowledge of the vector species in the region. Alternative vector control tools that target outdoor-resting mosquitoes, partially zoophilic mosquitoes, and mosquitoes that feed in the early evening are also vital to the success of malaria elimination in Latin America. Risk mapping can aid the deployment of alternative tools such as attractive toxic sugar bait (ATSB), which target outdoors vector populations [97]. The ATSB method works through bait stations or spraying an attractive sugar solution containing an oral toxin on spots of vegetation in order to kill mosquitoes that feed on it [98–102].

## Understanding the influence of human and environmental disruptions

The terrain of Latin America is also a major determinant of applicable vector control interventions. Considering that many countries contain extensive wetlands, and flooding is frequent during the rainy season, vector management through larval control becomes difficult. It is important to note that the rainy season is typically not the time of peak transmission because the larvae and pupae are swept away in currents; whereas at the end of the wet season/beginning of the dry, is when transmission occurs owing to the slower flow rates in rivers, streams, and wetlands [103]. Larval control is often difficult in slums where some of the houses are built on stilts in water because of the cost, and possible harmful effects on non-target organisms, the environment or humans [104]. Locating breeding sites where larval control could be successful is also challenging especially in isolated areas in the Amazon where the logistics of locating and treating individual breeding sites may preclude control directed at the larval stage [103]. Despite these challenges, larval control with microbial larvicides is effective, particularly in small clearly defined larval habitats, like stagnant water bodies, storm drains or inundated forest floors and insect growth regulators or OP larvicides are effective in clearly defined large water bodies as evidenced in studies in Central America [105, 106], Ecuador [10] and Peru [106].

Remote sensing has been extensively used to characterize location and distribution of larval habitats and direct target efforts towards mosquitoes at the larval stage. A number of vector studies have used RS to measure and identify aquatic macrophytes (AMs), which are found along the shallow margins of water bodies [107–109]. These AMs provide sources of information to identify where within water bodies and wetlands interventions such as larval control should be targeted. For instance, Rejmankova et al. [67, 108] used Système Probatoire d’Observation de la Terre (SPOT) images to identify and examine marshes in Belize, which contained AMs which serves as larval habitats for *An. albimanus* [67], *Anopheles vestitipennis* and *Anopheles punctimacula* [108] respectively. Roberts et al. [107] also used multispectral SPOT XS images containing thermal bands essential for mapping vegetation to predict presence of *An. pseudopunctipennis* in central Belize. Samson et al. [70] used a RapidEye image of the northern provinces of Haiti pre (2010) and post (2013) the earthquake to devise and implement a larval sampling strategy in the area.

Environmental changes, whether by humans or nature plays an important role in vector distribution and malaria control. In many parts of Latin America, increased malaria incidence is associated with land use changes. Land conversion for subsistence agriculture, pasture and livestock production [110], and infrastructural development e.g. dam construction such as the Belo Monte [111], in the Amazon basin lead to widespread deforestation [112]. The environmental alterations create larval habitats for specific anopheline larvae development [113] e.g. dams create stagnant water which serve as ideal breeding habitats [60] that will likely increase risk of malaria transmission in the near future. In their study, Taddei et al. [114] found *An. darlingi* in 13 out of 14 altered environments in the Brazilian Amazon whereas none was found in 5 unaltered areas. Vittor et al. [115] also observed that *An. darlingi* biting rates in deforested areas was 278 times those of forest areas in the Peruvian Amazon. So, as long as regulations on deforestation in the Amazon basin are not fully enforced, illegal activities such as gold mining and cacao cultivation remain unchecked, unplanned urbanization increases and more land acquisitions for agriculture and infrastructural developments continue, vector ecology will keep changing [110, 112–114, 116].

A huge problem remains in frontier areas along forest boundaries in Brazil where deforestation and extractive activities (mining, agriculture, logging, etc.) occur [117]. These create frontier settlements which favour human clustering close to vector habitats [117, 118], leading to ‘frontier malaria’ which are mapped through risk mapping methodologies [56, 117–119]. The Amazon is also porous so many extractive activities such as mining and logging take place without government sanction. The loggers and miners engaged in extractive activities are a focal point for transmission and dispersal of parasites within and between Amazonian countries. By mapping forest disturbance at small scales, elimination can be advanced because such disturbances are frequently associated with extractive activities where transmission is concentrated. Conflicts are also rife in these frontier zones in Brazil [120] as well as in other parts of the region such as Colombia [121]. These conflicts have destabilized communities, caused disruptions in government services (e.g. health care), and forced people to move from their homes, where they are likely to come into more contact with vectors. Thus, civil conflict is a risk factor that bears mapping. Data on measures that can serve as proxies for civil conflicts such as number of internally displaced persons at lower geographies e.g. municipalities can be potentially invaluable source of information to improve assessment of risk and risk maps. Not only can remote sensing address these risk hotspots, but efficacy mapping could be used to understand where investment in control efforts is falling short for whatever reason (i.e., lack of political will, corruption, and civil conflict). Remote sensing from satellites has played an important role in facilitating understanding of how land use land-cover (LULC) changes relate to malaria, particularly in the Amazon [122, 123]. The methodology has been used to associate deforestation [124, 125] and the environment [126] with malaria and its vectors. In a study conducted in Mancio Lima, Brazil, Olson et al. [124] showed through geographical and statistical analyses that a 4.3 % change in deforestation in the county between 1997 and 2000 was associated with 48 % rise in malaria incidence. Studies by Conn et al. [127] and Moreno et al. [128] suggests that human interference may foster the presence of *An. marajoara* while Vittor et al. [115, 125] observed that environmental changes may propagate spread of *An. darlingi*. Thus, small changes in forest cover can lead to major consequences and these changes can be assessed systematically through time using remote sensing from satellite and aircraft.

## Conclusion

**Fig. 2:**
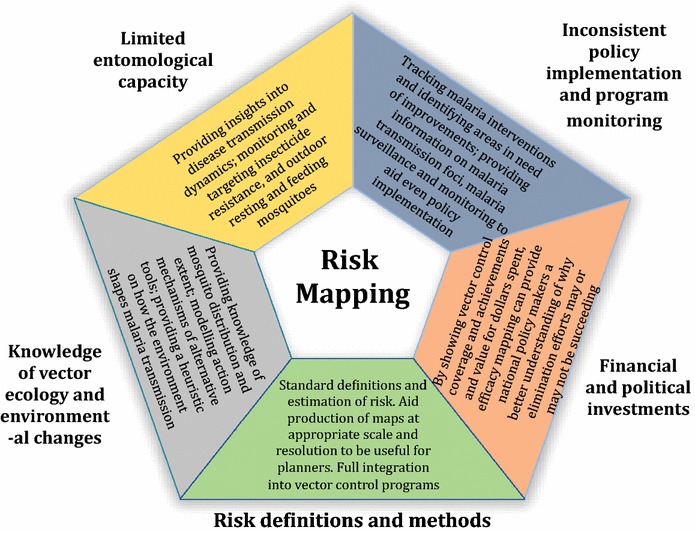
Recommendations for locally tailored vector control using risk mapping methodologies

National Malaria Control Programmes in Latin America have made huge progress in malaria control but more effort is needed to achieve elimination (Fig. 2). While elimination appears more feasible today than a decade ago, continuing with the current state of knowledge and operational system in vector control may delay implementation. A sure way of advancement is to strengthen aspects of the IVM programmes and policies where some NMCPs are still struggling.

- Evidence-based vector control is a fundamental tenet of the IVM, which cannot be carried out without strong entomological capacity. This entomological capacity can be strengthened through risk mapping methodologies which provides insights into disease transmission dynamics, can help monitor and target insecticide resistance and provide much needed spatial information on outdoor and early-biting mosquitoes. As such, investments in risk mapping technologies for entomological research are imperative as are maps that depict where further investment in capacity is needed.
- The need to implement and monitor IVM policies consistently across board can be met through applying risk-mapping technologies. Integrated information systems and modelling strategies can track malaria interventions and identify areas in need of improvements. Information on human populations, vector behaviour and distribution, malaria transmission foci and updates on malaria surveillance and monitoring can be readily available through risk mapping. When fully integrated in vector control programmes, these technologies can accelerate the drive towards elimination.
- The definition and estimation of malaria risk need to be standardized so that risk maps can be comparable across countries. The maps also need to possess the appropriate scale and resolution for planners to target areas in greatest need of control measures. Since elimination has to occur locally, large scale (1:5000 or greater) and high-resolution (30 m or less) risk maps are needed to guide IVM implementation and elimination efforts on the ground. Risk mapping should also become an integral part of the information system so that targeted vector control can be conducted. Once integrated, risk-mapping methodologies will aid decision-making, disease risk management and allow more effective allocation of resources for malaria control.
- Brazil’s policies and sustained investment in early detection and treatment in isolated areas demonstrate that government commitment to elimination is feasible and that declines in incidence can be achieved even in geographically isolated parts of the Amazon. However, linking these efforts (i.e., matching investment maps to actual outcomes) to risk mapping (through efficacy mapping) can provide national policy-makers a better understanding of why elimination efforts are succeeding in some areas and not in others. This “risk mapping” frontier could greatly enhance elimination efforts in Latin America and elsewhere. Hence, governments need to take ownership by increasing domestic funding for malaria control and investing in research that advances the use of risk-mapping methodologies in vector control programmes.
- In order to bridge knowledge gaps in distribution and behaviour of Latin American vectors, extensive applications of risk-mapping techniques, particularly SDMs should be encouraged, as SDMs such as MaxEnt are freely available and relatively easy to use to map probability of vector or disease presence. The methodologies can also help improve and facilitate the development of alternative vector control strategies.
- Gaining a better understanding of the influence of human and environmental disruptions in malaria epidemiology and vector ecology will help direct future projects and minimize the impact of actions in the disease dynamics. Remote sensing from satellites in particular provides a consistent source of environmental information that enhances understanding of how risk may change through time as a function of changes in vegetation cover, hydrology, and coverage of AMs.
- This review has identified the many ways that risk mapping and modelling may address the constraints to malaria elimination in Latin America as well as highlighted the limitations and factors precluding its widespread use. As such, incorporating risk mapping methodologies as a fundamental part of vector control programmes in Latin America could help guide malaria control interventions, potentially making malaria elimination in the region more feasible.

## Abbreviations

API: Annual Parasite Index
WHO: World Health Organization
IVM: Integrated Vector Management
LLIN: Long-lasting insecticidal nets
IRS: Indoor Residual Spraying
BG: Bayesian Geo-statistics
DDT: Dichlorodiphenyltrichloroethane
OP: Organophosphates
IR: Insecticide Resistance
PAHO: Pan American Health Organization
GIS: Geographic Information Systems
RS: Remote Sensing
MoH: Ministry of Health
SIVEP: Sistema de Informação de Vigilância Epidemiológica
EMMIE: Eliminación de Malaria en Mesoamerica y la Isla Española
GFATM: Global Fund for Aids, Tuberculosis and Malaria (GFATM)
AM: Aquatic Macrophytes
SDM: Species Distribution Models
MaxEnt: Maximum Entropy
BRT: Boosted Regression Trees
ATSB: Attractive Toxic Sugar Baits
LULC: Land use and land cover
SPOT: Systeme Probatoire d’Observation de la Terre
MCDA: Multi Criteria Decision Analysis
